# Are *old-old *patients with major depression more likely to relapse than *young-old *patients during continuation treatment with escitalopram?

**DOI:** 10.1186/1471-2318-11-2

**Published:** 2011-01-14

**Authors:** Constantine G Lyketsos, Emmanuelle Weiller, Cornelius Katona, Phillip Gorwood

**Affiliations:** 1 Department of Psychiatry, School of Medicine, Johns Hopkins University and Johns Hopkins Bayview Medical Center, Baltimore, MD, USA; 2 H. Lundbeck A/S, Copenhagen, Denmark; 3 Department of Mental Health Sciences, University College London, UK; 4 INSERM U894 (Centre de Psychiatrie et Neurosciences), Sainte-Anne Hospital, Paris, France

## Abstract

**Background:**

Escitalopram has shown efficacy and tolerability in the prevention of relapse in elderly patients with major depressive disorder (MDD). This *post-hoc *analysis compared time to relapse for *young-old *patients (n = 197) to that for *old-old *patients (n = 108).

**Method:**

Relapse prevention: after 12-weeks open-label treatment, remitters (MADRS ≤12) were randomised to double-blind treatment with escitalopram or placebo and followed over 24-weeks. Patients were outpatients with MDD from 46 European centers aged ≥75 years (*old-old*) or 65-74 years of age (*young-old*), treated with escitalopram 10-20mg/day. Efficacy was assessed using the Montgomery Åsberg Depression Rating Scale (MADRS).

**Results:**

After open-label escitalopram treatment, a similar proportion of *young-old *patients (78%) and *old-old *patients (72%) achieved remission. In the analysis of time to relapse based on the Cox model (proportional hazards regression), with treatment and age group as covariates, the hazard ratio was 4.4 for placebo *versus *escitalopram (χ^2^-test, df = 1, χ^2^= 22.5, p < 0.001), whereas the effect of age was not significant, with a hazard ratio of 1.2 for *old-old *versus *young-old *(χ^2^-test, df = 1, χ^2 ^= 0.41, p = 0.520). Escitalopram was well tolerated in both age groups with adverse events reported by 53.1% of *young-old *patients and 58.3% of *old-old *patients. There was no significant difference in withdrawal rates due to AEs between age groups (χ^2^-test, χ^2 ^= 1.669, df = 1, p = 0.196).

**Conclusions:**

*Young-old *and *old-old *patients with MDD had comparable rates of remission after open-label escitalopram, and both age groups had much lower rates of relapse on escitalopram than on placebo.

## Background

Older adults with depression often have several chronic disorders and are more treatment-resistant [1]. These patients also have a higher risk of medication side effects, due to co-morbid medical conditions and age-associated changes in organ function. Moreover, a higher risk for drug-drug interactions may cause them to terminate treatment prematurely.

Second generation antidepressants [selective serotonin reuptake inhibitors (SSRIs) and serotonin-noradrenaline reuptake inhibitors (SNRIs)] are recommended as first-line treatments in clinical practice guidelines and are most commonly prescribed for depressive disorders in older patients [2]. Nevertheless, until 2003, only one large placebo-controlled trial of a non-tricyclic antidepressant, marketed in the US, in outpatients 60 years of age or older with major depressive disorder had been published [3]. The first trials of second-generation antidepressants, including a large study of patients aged 75 years and above treated with citalopram, reported no advantage over placebo [4-6] or small drug-placebo differences [3,7], hence the clinical value of these agents in treating older depressed adults was uncertain.

A later meta-analysis of 10 randomised placebo-controlled trials in depressed patients aged 60 years and older concluded that antidepressants are more effective than placebo, although effects were modest and variable [8]. A major limitation was that the average age of patients in these trials ranged from 60 to 72 years, with only a small number over 75. Late-life spans a broad age range and can be divided into *young-old *(60-74 years), and *old-old *(≥75 years). Among the *old-old*, antidepressant treatment may be especially complicated because of the high frequency and severity of co-morbid conditions, such as cognitive impairment or heart disease.

In addition to being well tolerated as an antidepressant in older persons [4,5,9-11], escitalopram is effective in preventing relapse of MDD in patients ≥65 years [12]. The latter study, as well as open-label data, suggests that escitalopram is safe and well tolerated in the long-term treatment of older patients with MDD [9].

In order to compare the efficacy and safety of escitalopram in the *old-old *with the *young-old*, we undertook a secondary analysis of a published clinical trial [12]. The primary aim was to compare the effect of escitalopram in preventing relapse of MDD among *young-old *and *old-old *patients. A secondary objective was to compare the initial response to open-label escitalopram in these two age groups. We hypothesized that *old-old *and *young-old *patients on escitalopram would have comparable benefits with regard to prevention of relapse and initial remission.

## Methods

The original study was conducted in 46 centers in 7 European countries from October 2003 to May 2005, in accordance with the principles of *Good Clinical Practice *[13] and the *Declaration of Helsinki *[14] applicable at the time of the study. The study was approved by the relevant local ethics committees and all patients gave written informed consent for participation. The methods and main results of the principal study have been described [12].

### Design Overview

**Figure 1:**
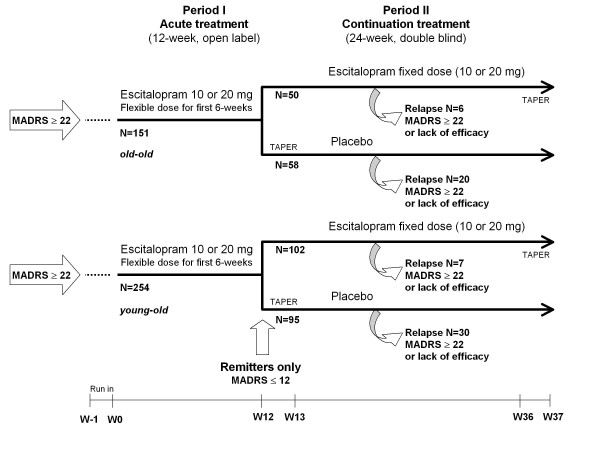
**Study design**.

This relapse prevention study started with a 12-week open-label treatment period that was followed by a 24-week, randomised, double blind treatment period (Figure 1). During the initial open-label period, outpatients aged at least 65 years received escitalopram 10mg/day during the first week; the dose could be increased to 20mg/day at Week 2, based on the clinical judgement of the investigator. Subsequently the dose remained constant for the remaining open label period. At the end of the 12-week open-label period, patients who achieved remission, defined by a Montgomery Åsberg Depression Rating Scale [15] (MADRS) score ≤12, were eligible for randomisation to either continue escitalopram at their last fixed dose of 10 or 20mg/day, or to switch to placebo in a 1:1 ratio. Patients randomised to placebo who were on 20 mg/day escitalopram during the open period received 10 mg/day escitalopram for the first week (Week 13) before receiving placebo for the remainder of the study. Non-remitters left the study and were treated at the physician's discretion.

During the 24-week double-blind period (until taper at Week 36), study investigators regularly evaluated relapse symptoms. Relapse was defined either as a MADRS total score ≥22 at a single visit, or an unsatisfactory treatment effect (lack of efficacy), as judged by the local investigator. In order to capture all patients who could be considered to have relapsed, this definition was extended in a sensitivity analysis to include patients who met the DSM-IV criteria for MDE or who attempted suicide. Patients who relapsed were withdrawn from the study and were contacted for a safety follow-up assessment 4 weeks later.

### Patients

Psychiatrists recruited patients in private practice or hospital outpatient clinics. Those eligible for study were outpatients with a primary diagnosis of MDD (assessed by psychiatrists using the Mini International Neuropsychiatric Interview) [16], moderate or severe, according to DSM-IV-TR criteria [17], and who gave informed consent. Inclusion and exclusion criteria are described in detail by Gorwood et al. [12]. Patients were required to have a MADRS score ≥22 and an MMSE score ≥24 at entry. Patients with severe or unstable medical co-morbid diseases were excluded.

### Medication treatment

Study medications were tablets for oral administration of identical appearance, taste and smell. The oxalate salt of escitalopram, or a placebo, was used in the tablets. After the open-label period, eligible patients were assigned to escitalopram or placebo according to a computer-generated randomisation list drawn up by H. Lundbeck A/S. Details of the randomisation were unknown to any of the investigators and were contained in a set of sealed opaque envelopes. At each study center, sequentially enrolled patients were assigned the lowest randomisation number available in blocks of four. All study personnel and participants were blinded to treatment assignment for the duration of the study.

### Assessments

After baseline assessment of efficacy measures, efficacy and tolerability parameters were assessed after 2, 4, 6, 8, and 12 weeks of open-label treatment. For patients randomised to double-blind treatment, efficacy and tolerability parameters were assessed 1, 2, and 4 weeks after randomisation, and then every 4 weeks until the last dose of double-blind treatment (Week 24 of double blind, also Week 36 of the full study).

Efficacy assessments at baseline and each follow-up visit included the MADRS, Clinical Global Impression - Severity of illness (CGI-S) and Clinical Global Impression-Improvement of illness (CGI-I) [18]. The primary analysis of efficacy was the time to relapse from the start date of double-blind treatment. Prior to the study, all investigators attended a joined MADRS and CGI rating session chaired by an experienced research psychiatrist.

The tolerability and safety evaluations were based on spontaneously reported adverse events (AEs), vital signs, body weight, and physical examination.

### Statistical Analysis

To provide more clinically useful information in the age contrast during the open label period, patient response was pre-defined as at least 50% improvement from baseline in the MADRS total score, as was remission (MADRS ≤12). Complete remission (MADRS ≤5) was defined *post hoc*. All efficacy analyses in the double-blind period were conducted on the modified intent-to-treat (ITT) dataset, consisting of all randomised patients who took at least one dose of trial medication in the double-blind treatment period, using the last observation carried forward (LOCF) approach to impute missing data. Comparisons between age groups during the open-label period were performed using analysis of covariance (ANCOVA) with age group and country as factors, and with the score at randomisation as a covariate.

The pre-defined primary efficacy analysis [12] used a two-tailed log-rank test to compare the time to relapse for patients treated with escitalopram *versus *placebo, using SAS version 9.1 as statistical software. In addition, Kaplan-Meier survival curves were produced and Cox proportional hazards models were estimated to assess the effect of several variables on time to relapse. A χ^2^-test was used to compare the crude proportions of relapsed patients. In the current study, the focus was on the effect of age - comparing the *old-old *(patients aged ≥75 years) *versus *the *young-old *(65 to 74 years), using a Cox model and logistic regression, with treatment and age group as covariates. The appropriateness and robustness was studied by investigating the possible effect of country and of country-by-treatment interaction. The results from analyses with center as a covariate were in line with that of the primary analysis and revealed no "outliers" among countries or centers.

## Results

### Patient Characteristics

Of the 405 patients who received open-label escitalopram, 254 were aged 65-74 years *(young-old) *and 151 were ≥75 years of age *(old-old*). The baseline characteristics of these two groups were similar, apart from differences in mean age at MDD onset. Also, the *old-old *group had a slightly higher percentage of women, slightly more ongoing general medical diseases per patient, and more patients with a first episode of MDD (Table 1). There were no clinically relevant differences in the severity of depression as measured by MADRS or CGI-S between *young-old *and *old-old *patients (Table 1).

**Table 1:** Baseline patient characteristics

	**Patients aged 65-74 years**^**a**^ (n = 254)	Patients aged ≥75 years (n = 151)
Mean age in years (SD)	69 (3)	79 (4)
Sex (n, % women)	193 (76%)	120 (79%)
BMI in kg/m^2 ^(SD)	26.5 (4.2)	25.8 (4.0)
Mean duration of current MDE in weeks (SD)	16.3 (15.1)	16.6 (16.5)
Mean age at MDD onset in years (range)	62 (18-74)	71 (12-90)
First episode (n, %)	67 (26.4%)	51 (33.8%)
Co-morbid diseases/patient (mean ± SD)	3.1 (2.1)*	3.7 (2.3)
MADRS total score (mean ± SD)	31.1 (5.0)	30.9 (4.1)
CGI-S (mean ± SD)	4.9 (0.7)	4.8 (0.7)

^a ^all patients were at least 65 years of age

BMI: body mass index, CGI-S: Clinical Global Impression - Severity of illness, MADRS: Montgomery-Åsberg Depression Rating Scale, MDE: major depressive episode; SD: standard deviation * p < 0.05 versus patients aged ≥75 years

### Comparison by age in the 12-week open label period

#### Withdrawals

**Figure 2:**
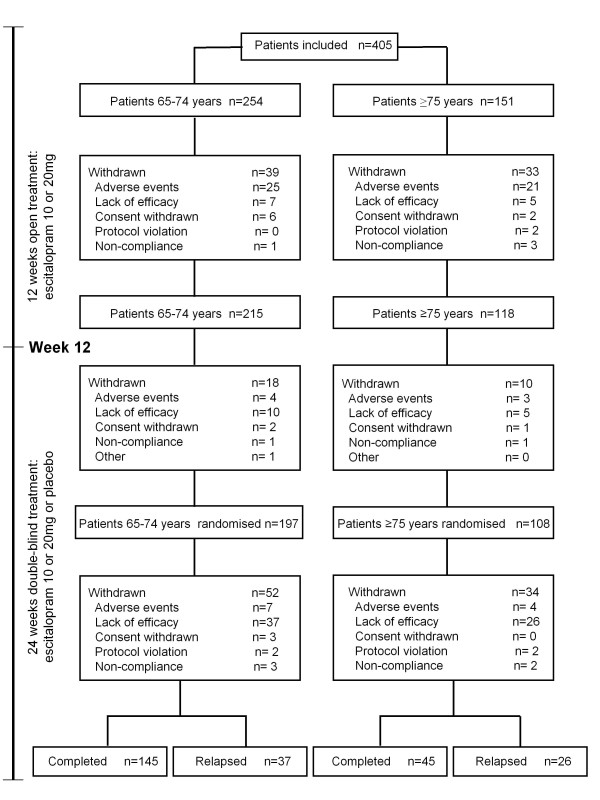
**Patient disposition for the open label period and the randomised double-blind period of the study**. AEs: adverse events, CW: consent withdrawn, LoE: lack of efficacy, NC: non-compliance, PV: protocol violation.

During the open-label period, 39 out of 254 *young-old *patients (15.4%) withdrew, 25 due to AEs (9.8%) (Figure 2). For *old-old *patients, 33 out of 151 patients (21.9%) withdrew during the 12-week open-label period, 21 due to AEs (13.9%). The difference in withdrawal rates was not statistically significant between the two age groups (logistic regression, Wald χ^2 ^= 1.8484, df = 1, p = 0.174). There was also no significant difference between age groups in withdrawal rates due to AEs (chi-square test, χ^2 ^= 1.55, df = 1, p = 0.212).

#### Efficacy

Of the 254 *young-old *patients entering open-label treatment, 215 achieved remission (78%) and 197 were randomly assigned to treatment with escitalopram (n = 102) or placebo (n = 95) (Figure 2). The *young-old *patients had a mean baseline MADRS total score of 31.1 ± 5.0 at entry, which decreased over time to 9.0 ± 9.5 (LOCF). Similarly, of the 151 *old-old *patients entering the open-label period, 118 achieved remission (72%) and 108 were randomly assigned to treatment with escitalopram (n = 50) or placebo (n = 58) (Figure 2). These patients had a mean baseline MADRS total score of 30.9 ± 4.1 at inclusion, which decreased over time to 10.9 ± 9.8 (LOCF).

A similar proportion of *young-old *(83.1%, n = 211) and *old-old *(77.5%, n = 117) patients responded (ITT, LOCF, Fisher's Exact test, p = 0.339,) or achieved remission (*young old: *81.9%, n = 208; *old-old *75.5%, n = 114) (ITT, LOCF, Fisher's Exact test, p = 0.128) by week 12. A significantly higher proportion of *young-old *patients achieved complete remission (MADRS ≤5) at week 12 (*young old: *48.4%, n = 123; *old-old *35.1%, n = 53) (Fisher's Exact test, p = 0.010) (ITT, LOCF). However, decreases from baseline to week 12 in the mean MADRS score were not significantly different between the two age groups: -22.0 (SD = 17.8) points for patients aged 65-74 years, and -20.3 (SD = 14.1) points for patients ≥75 years; LOCF, p = 0.084 (ANCOVA, using age group as factor, and baseline score as covariate). The mean score of individual MADRS items for patients completing open-label treatment indicates that the *old-old *patients had slightly more residual symptoms at the end of the open label phase of treatment, particularly in the form of persistent inner tension and sleep disturbance (data not shown).

#### Tolerability

AEs were reported by 135 (53.1%) of *young-old *patients (n = 254) and 88 (58.3%) of *old-old *patients (n = 151). Treatment-emergent AEs with an incidence ≥5% in either age group that occurred during the open-label period were nausea, headache, dizziness, diarrhoea, fatigue, hyperhidrosis and insomnia. The difference by age was statistically significant only for hyperhidrosis [15 (5.9%) *versus *2 (1.3%), respectively; p = 0.0372, Fisher's Exact test] and diarrhoea [17 (6.7%) *versus *3 (2.0%), respectively; p = 0.0348, Fisher's Exact test]. In almost all cases, spontaneously reported AEs were mild to moderate. There was no significant difference in withdrawal rates due to AEs between age groups (chi-square, χ^2 ^= 1.669, df = 1, p = 0.196). The most common AEs leading to withdrawal for *old-old *patients (n = 151) were nausea (14 patients), anxiety (7 patients), and depression (5 patients) compared to nausea (9 patients), anxiety (3 patients), and depression (3 patients), for *young-old *patients. Of the 21 *old-old *patients who withdrew due to an AE, there were 19 (out of 100) aged from 75 to 79 years, 2 (out of 39) aged from 80 to 84 years, and 3 (out of 12) aged 85 years or above. *Old-old *patients had a significantly higher incidence of serious adverse events (SAEs) (12 out of 151 patients, 7.9%) than *young-old *patients (5 out of 254 patients, 2.0%) (Fisher's Exact test, p = 0.008). A 75-year-old man with a *possibly-related *SAE was hospitalized for 27 days due to anxiety and suicidal ideation and subsequently recovered.

### Comparison by age in the 24-week randomized continuation period

#### Withdrawals

Of the patients continuing into the double-blind period of the study, 74 (40 escitalopram-treated and 34 placebo-treated patients) completed the study, corresponding to completion rates of 80% (escitalopram) and 59% (placebo). The overall withdrawal rate excluding relapses, was comparable for both age groups (7.6% for *young-old *versus 7.4% for *old-old*): 5.9% for *young-old *patients treated with escitalopram (total of 102 patients, 6 withdrawals not due to relapse), and 9.5% for *young-old *patients treated with placebo (total of 95 patients, 9 withdrawals not due to relapse). For *old-old *patients, the overall withdrawal rate excluding relapses, was 8.0% for patients treated with escitalopram (total of 50 patients, 4 withdrawals not due to relapse), and 6.9% for patients treated with placebo (total of 58 patients, 4 withdrawals not due to relapse).

#### Relapse prevention

*Old-old *patients randomised to double-blind treatment had mean MADRS total scores of 5.8 (both treatment groups) at week 12, compared to 5.0 for *young-old *patients. For *old-old *patients, the proportion of patients who relapsed within 24 weeks was significantly higher on placebo (34.5%; 20 out of 58 patients) than on escitalopram (12.0%; 6 out of 50 patients) (chi-square test, χ^2 ^= 7.426, df = 1, p = 0.006). For *young-old *patients, the proportion of patients who relapsed within 24 weeks was also significantly higher on placebo (31.6%; 30 out of 95 patients) than on escitalopram (6.9%; 7 out of 102 patients) (chi-square test, χ^2 ^= 19.698, df= 1, p < 0.001).

**Figure 3:**
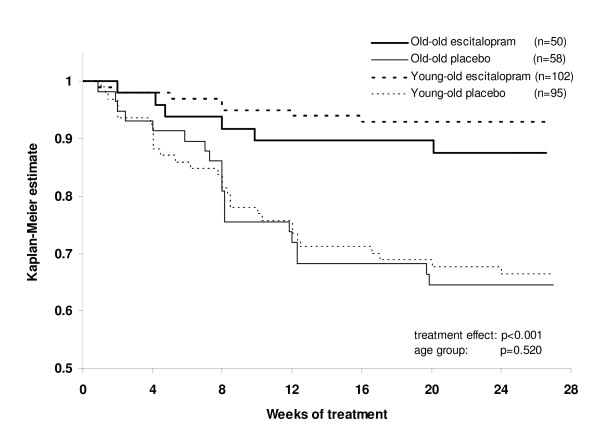
**Kaplan Meier survival analysis of relapse over 24 weeks**. Time to relapse (Cox model, with treatment and age group as covariates) showed significant advantage for treatment with escitalopram compared to placebo, with an estimated hazard ratio of 4.4 (chi-square test, df = 1, χ^2 ^= 22.5, p < 0.001), whereas the effect of age group was not significant, with an estimated hazard ratio of 1.2 for *old-old versus young-old *(chi-square test, χ^2 ^= 0.41, df = 1, p = 0.520).

When comparing age groups, stratified by treatment, there was no statistically significant difference in time to relapse between *young-old *patients versus *old-old *patients after randomisation to placebo (Figure 3, log-rank test comparing the two placebo groups, χ^2 ^= 0.0404, df = 1, p = 0.841) or to escitalopram (Figure 3, log-rank test comparing the two escitalopram groups, χ^2 ^= 1.1497, df = 1, p = 0.284). In the analysis of time to relapse, based on the Cox proportional hazard model, with treatment and age group as covariates, the estimated hazard ratio for relapse on placebo *versus *escitalopram was 4.4 (95% confidence interval: 2.4 to 8.1; chi-square test, χ^2 ^= 22.5, df = 1, p < 0.001), whereas the effect of age group was not significant, with a hazard ratio of 1.2 (95% confidence interval: 0.7 to 1.9, chi-square test, χ^2 ^= 0.41, df = 1, p = 0.520) for *old-old versus young-old *patients. The proportion of *young-old *patients who relapsed based on the investigator's judgment was 29.7% (11/37) compared to 30.8% (8/26) of *old-old *patients. The estimated hazard ratio for relapse on placebo compared to escitalopram was 5.4 for *young-old *patients and 3.2 for *old-old *patients. The proportion of patients who remained in remission (MADRS ≤12) to the end of the full study was higher on escitalopram (89% for *young-old *patients *versus *86% for *old-old *patients) compared to placebo (61% for *young-old *patients *versus *57% for *old-old *patients) (LOCF). In the sensitivity analysis, where the relapse criteria were extended to include patients who met the DSM-IV criteria for MDE, an additional 2 patients relapsed, both in the placebo group.

#### Tolerability

During this period, AEs led to the withdrawal of 5 *young-old *patients and 4 *old-old *patients. The most commonly reported AEs in *young-old *patients were headache (8.1%), dizziness (5.6%), and diarrhoea (5.1%), and in *old-old *patients, headache (3.7%), dizziness (6.5%), hypertension (4.6%) and nasopharyngitis (4.6%). SAEs were reported by 7 *young-old *and 6 *old-old *patients. A 74-year-old man had a *probably-related SAE *during treatment with placebo was hospitalised for 27 days due to relapse of depression and was withdrawn. An 85-year-old woman with a *possibly-related *SAE during treatment with escitalopram was hospitalised with anaemia due to a gastric mucosal ulcer and subsequently recovered.

## Discussion

The results of these *post-hoc *analyses indicate that *young-old *and *old-old *patients with MDD have comparable rates of remission after open-label escitalopram, and both age groups have statistically significantly lower rates of relapse on escitalopram than on placebo.

To date, the few studies of pharmacological treatment in this age group have primarily or exclusively included patients in residential settings [19-21]. However, most depressed patients aged at least 75 years live in the community. They tend to have less cognitive impairment than nursing home residents [6]. Open-label treatment with escitalopram was comparably effective in reducing depression in *young-old *as in *old-old *patients, consistent with the results of the meta-analysis of Gildengers et al. [22]. Notably, a significantly higher percentage of *young-old *patients achieved complete remission, likely related to the slightly higher level of symptoms at entry in the *old-old *patients, particularly for MADRS items 3 to 8. The response rates (77% and 83%) in both age groups were greater than those reported in 2 placebo-controlled trials of escitalopram in elderly patients (46% response) [4,5], in which escitalopram failed to separate from placebo. Sneed et al. [23] also found higher response rates in patients in comparator trials (60% response) compared with placebo-controlled trials (46% response). Hence, in the absence of a placebo control, these rates of remission should not be construed as being entirely related to escitalopram treatment. In addition to the clinician-rated MADRS, the geriatric depression scale was also used in the original study [12]. This patient self-rating scale was only assessed at baseline, at randomisation and at last assessment. It is a validated scale for the assessment of the health status of older people, but does not directly assess symptoms of depression.

The pattern of AEs was similar in both age groups, and to that previously seen with escitalopram in younger adults [24]. Based on the incidence of adverse events and withdrawals due to adverse events, *old-old *patients tolerated treatment with escitalopram equally well as *young-old *patients. During the 24-week relapse prevention period, escitalopram was significantly more efficacious than placebo.

This study's main strength is that, as a result of the high remission rates during the open-label escitalopram phase, there were relatively large numbers of patients in both the *young-old *and *old-old *groups at the point of randomisation, giving the study satisfactory statistical power to detect differences in relapse rate and pattern between them. The analysis by age group was, however, retrospective, and its inclusion and exclusion criteria to some extent limit the generalizability of its findings to the depressed *old-old *population. A further limitation of the study is that we did not investigate in detail the effect of medical co-morbidities on outcomes. As Reynolds et al. [25] note, the number and severity of co-morbid medical illnesses moderate depression recurrence in older old depressed patient. Since medical co-morbidities were comparable across the randomization groups (Table 2), it is unlikely that medical co-morbidity influenced any treatment benefit from escitalopram.

**Table 2:** Co-morbid medical conditions at baseline with a prevalence ≥5% in either age group

Diagnosis*	Patients aged 65-74 years (n = 254)	Patients aged ≥75 years (n = 151)
Hypertension	129 (50.8%)	86 (57.0%)
Myocardial ischemia	31 (12.2%)	34 (22.5%)
Localised osteoarthritis	19 ( 7.5%)	16 (10.6%)
Osteoarthritis	10 ( 3.9%)	16 (10.6%)
Hypercholesterolaemia	26 (10.2%)	15 ( 9.9%)
Insomnia	23 ( 9.1%)	15 ( 9.9%)
Osteoporosis	23 ( 9.1%)	15 ( 9.9%)
Diabetes mellitus	12 ( 4.7%)	12 ( 7.9%)
Diabetes mellitus non-insulin-dependent	20 ( 7.9%)	11 ( 7.3%)
Varicose vein	13 ( 5.1%)	10 ( 6.6%)
Coronary artery disease	10 ( 3.9%)	9 ( 6.0%)
Gastritis	7 ( 2.8%)	9 ( 6.0%)
Cardiac failure	2 ( 0.8%)	9 ( 6.0%)
Cataract	3 ( 1.2%)	8 ( 5.3%)
Back pain	17 ( 6.7%)	6 ( 4.0%)
Hypothyroidism	16 ( 6.3%)	5 ( 3.3%)

* These conditions were judged as stable by study physicians, as required by the study inclusion criteria

## Conclusions

The study has clear and relevant implications for clinical practice. The slightly higher percentage *of young-old *patients achieving complete remission during the open-label phase probably reflects the somewhat higher burden of symptoms that *old-old *patients have at the start of acute treatment. The comparable (and very low) incidence of adverse events and of adverse event-related withdrawals in *old-old *and *young-old *patients in both phases of the study suggests that escitalopram is well-tolerated by *old-old *depressed patients. Although slightly fewer *young-old *patients on escitalopram relapsed (6.9% vs 12% in the *old-old *group), the hazard ratio was only slightly larger (1.2). It is therefore clear that escitalopram is highly effective (compared with placebo) in preventing depressive relapse in *old-old *patients who remit during acute escitalopram treatment, as shown by the hazard ratio in this sub-group.

## Competing interests

CK has received honoraria from, and have conducted clinical research supported by H. Lundbeck A/S. PG is employed by University Paris Descartes and has received grants from Eli Lilly and Servier, honoraria from Bristol-Myers Squibb, Eli Lilly, Lundbeck, Servier and UCB-Pharma, and participated in advisory boards for Janssen, Lundbeck, Servier and Wyeth. CGL has received grant support (research or CME) from NIMH, NIA, Associated Jewish Federation of Baltimore, Weinberg Foundation, Forest, Glaxo-Smith-Kline, Eisai, Pfizer, Astra-Zeneca, Lilly, Ortho-McNeil, Bristol-Myers, Novartis. He is a consultant/advisor to Astra-Zeneca, Glaxo-Smith Kline, Eisai, Novartis, Forest, Supernus, Adlyfe, Takeda, Wyeth, Lundbeck, Merz, Lilly, Genentech, Pfizer, and has received honoraria or travel support from Pfizer, Forest, Glaxo-Smith Kline, Health Monitor. EW is a fulltime employee of H. Lundbeck A/S.

## Authors' contributions

CK, EW, and PG contributed to the design of this study. All authors participated in interpretation of the data, drafting the manuscript and all read and approved the final manuscript. CK and EW supervised the statistical analyses, and all authors had full access to the data.

## Pre-publication history

The pre-publication history for this paper can be accessed here:

http://www.biomedcentral.com/1471-2318/11/2/prepub
